# Feedback Control in a General Almost Periodic Discrete System of Plankton Allelopathy

**DOI:** 10.1155/2014/824801

**Published:** 2014-01-23

**Authors:** Wenshuang Yin

**Affiliations:** Department of Mathematics, Hubei Minzu University, Enshi 445000, China

## Abstract

We study the properties of almost periodic solutions for a general discrete system of plankton allelopathy with feedback controls and establish a theorem on the uniformly asymptotic stability of almost periodic solutions.

## 1. Introduction

Allelopathy is first used by Hans (see [1]) in 1937, which is a biological phenomenon that is characteristic of any process involving secondary metabolites produced by some plants, algae, bacteria, and fungi which influences the growth and development of biological systems. There are many investigations which have been conducted to study the toxic effect on plankton allelopathy (see [2–9]). In this paper, we investigate the following system with feedback controls:

(1) xi(n+1)=xi(n)exp⁡⁡[ri(n)−aii(n)xi(n)−aij(n)xj(n)−bi(n)xi(n)xj(n)−ci(n)ui(n)],Δui(n)=−di(n)ui(n)+ei(n)xi(n), i,j=1,2;  i≠j.

Here *x*_*i*_(*n*) represent the densities of species *x*_*i*_ at the *n*th generation, *r*_*i*_(*n*) are the intrinsic growth rates of species *x*_*i*_ at the *n*th generation, *a*_*ii*_(*n*) measure the intraspecific effects of the generation of species *x*_*i*_ on their own population, and *a*_*ij*_(*n*) stand for the interspecific effects of the *n*th generation of species *x*_*i*_ on species *x*_*j*_; *b*_*i*_(*n*) are the rates of toxic inhibition of species *x*_*i*_ by species *x*_*j*_ and vice versa at the *n*th generation, Δ is the first-order forward difference operator Δ*u*_*i*_(*n*) = *u*_*i*_(*n* + 1) − *u*_*i*_(*n*), and *u*_*i*_(*n*) are the feedback control variables (*i*, *j* = 1,2; *i* ≠ *j*). Meanwhile, {*r*_*i*_(*n*)}, {*a*_*ij*_(*n*)}, {*b*_*i*_(*n*)}, {*c*_*i*_(*n*)}, {*d*_*i*_(*n*)}, and {*e*_*i*_(*n*)} are bounded nonnegative sequences such that

(2) $$\begin{array}{c} 0<r_{i}^{l}\leq r_{i}\left(n\right)\leq r_{i}^{u},\\ 0<a_{ij}^{l}\leq a_{ij}\left(n\right)\leq a_{ij}^{u},\\ 0<b_{i}^{l}\leq b_{i}\left(n\right)\leq b_{i}^{u},\\ 0<c_{i}^{l}\leq c_{i}\left(n\right)\leq c_{i}^{u},\\ 0<e_{i}^{l}\leq e_{i}\left(n\right)\leq e_{i}^{u},\\ 0<d_{i}^{l}\leq d_{i}\left(n\right)\leq d_{i}^{u}<1, \end{array}$$

for *i*, *j* = 1,2. Here, we use the notations *f* ^*u*^ = sup_*n*∈*ℤ*^+^_ *f*(*n*) and  *f* ^*l*^ = inf_*n*∈*ℤ*^+^_ *f*(*n*) for any bounded sequence {*f*(*n*)}. For the simplicity and convenience of exposition, throughout this paper we let *ℤ*, *ℤ* ^+^, and ℝ^+^ denote the sets of all integers, and nonnegative integers, nonnegative real numbers, respectively.

From the point of view of biology, we only consider {*x*_1_(*n*), *x*_2_(*n*), *u*_1_(*n*), *u*_2_(*n*)} are positive; that is,

(3) $$\begin{array}{c} x_{i}\left(n\right)>0,\;u_{i}\left(n\right)>0,\;i=1,2. \end{array}$$

For convenience, we give the following definition of persistence of system (1).

**Definition 1**

System (1) is said to be persistent if there exist positive constants *x*_i*_, *u*_i*_, *x*_i_*, and *u*_i_* which are independent of the solutions of system (1), such that any positive solution {*x*_1_(*n*), *x*_2_(*n*), *u*_1_(*n*), *u*_2_(*n*)} of system (1) satisfies

(4) $$\begin{array}{c} x_{i^{*}}\leq \underset{n\to +\infty }{\mathrm{liminf}}x_{i}\left(n\right)\leq \underset{n\to +\infty }{\mathrm{limsup}}x_{i}\left(n\right)\leq x_{i}^{*},\\ u_{i^{*}}\leq \underset{n\to +\infty }{\mathrm{liminf}}u_{i}\left(n\right)\leq \underset{n\to +\infty }{\mathrm{limsup}}u_{i}\left(n\right)\leq u_{i}^{*}, \end{array}$$

for *i* = 1,2.

The rest of this paper is organized as follows. We first give some preliminaries in Section 2. Next, we study the persistent property of (1) in Section 3 and the almost periodic property in Section 4. To the best of our knowledge, no work has been done for the discrete system (1) with feedback controls.

## 2. Preliminaries

In this section, we first introduce some definitions. For our purpose, we introduce the following notions with their properties. For convenience, *n*, *m*, *l*, *s*, *h*, *i*, *j*, *k*, *τ*, *δ*, and *r* in the sequel always denote integers, and the relevant intervals and inequalities are discrete ones. This section, devoted to investigate the persistent property of system (1). To do so, we need to make some preparations.

We first introduce some definitions.

**Definition 2 (see [10])**

A sequence *x* : *ℤ* → ℝ^*k*^ is called an almost periodic sequence if the *ε*-translation set of *x*

(5) $$\begin{array}{c} E\left\{\varepsilon ,x\right\}:=\left\{\tau \in \mathbb{Z}:\left|x\left(n+\tau \right)-x\left(n\right)\right|<\varepsilon \;\;\forall n\in \mathbb{Z}\right\} \end{array}$$

is a relatively dense set in *ℤ* for all *ε* > 0; that is, for any given *ε* > 0, there exists an integer *l*(*ε*) > 0 such that each discrete interval of length *l*(*ε*) contains a *τ* = *τ*(*ε*) ∈ *E*{*ε*, *x*} such that

(6) $$\begin{array}{c} \left|x\left(n+\tau \right)-x\left(n\right)\right|<\varepsilon \end{array}$$

for all *n* ∈ *ℤ*. *τ* is called the *ε*-translation number of *x*(*n*).

**Definition 3 (see [10])**

Let *f* : *ℤ* × *𝔻* → ℝ^*k*^, where *𝔻* is an open set in ℝ^*k*^. *f*(*n*, *x*) is said to be almost periodic in *n* uniformly for *x* ∈ *𝔻* or uniformly almost periodic for short if, for any *ε* > 0 and any compact set *S* in *𝔻*, there exists a positive integer *l*(*ε*, *S*) such that any interval of length *l*(*ε*, *S*) contains a *τ* for which

(7) $$\begin{array}{c} \left|f\left(n+\tau ,x\right)-f\left(n,x\right)\right|<\varepsilon \end{array}$$

for all *n* ∈ *ℤ* and all *x* ∈ *S*. *τ* is called the *ε*-translation number of *f*(*x*, *n*).

Next, we will introduce the following Lemmas.

**Lemma 4 (see [10])**

{*x*(*n*)} is an almost periodic sequence if and only if for any sequence {*h*_*k*_}⊂{*h*_*k*′_} such that *x*(*n* + *h*_*k*_) converges uniformly on *n* ∈ *ℤ* as *k* → *∞*. Furthermore, the limit sequence is also an almost periodic sequence.

**Lemma 5 (see [11])**

Assume that {*x*(*n*)} satisfies *x*(*n*) > 0 and

(8) $$\begin{array}{c} x\left(n+1\right)\leq x\left(n\right)\exp\left[a\left(n\right)-b\left(n\right)x\left(n\right)\right] \end{array}$$

for *n* ∈ *ℤ* ^+^, where *a*(*n*) and *b*(*n*) are nonnegative sequences bounded above and below by positive constants. Then

(9) $$\begin{array}{c} \underset{n\to +\infty }{\mathrm{limsup}}\;x\left(n\right)\leq \frac{1}{b^{l}}\exp\left(a^{u}-1\right). \end{array}$$

**Lemma 6 (see [11])**

Assume that {*x*(*n*)} satisfies

(10) $$\begin{array}{c} x\left(n+1\right)\geq x\left(n\right)\exp\left[a\left(n\right)-b\left(n\right)x\left(n\right)\right],\;n\geq n_{0}, \end{array}$$

and limsup_*n*→+*∞*_ *x*(*n*) ≤ x*, *b* ^*u*^ x* > *a* ^*l*^, *x*(*n*_0_) > 0, where *a*(*n*) and *b*(*n*) are nonnegative sequences bounded above and below by positive constants and *n*_0_ ∈ *ℤ* ^+^. Then

(11) $$\begin{array}{c} \underset{n\to +\infty }{\mathrm{liminf}}\;x\left(n\right)\geq \frac{a^{l}}{b^{u}}\exp\left(a^{l}-b^{u}x^{*}\right). \end{array}$$

## 3. Persistence

In this section, we will establish sufficient conditions for the persistence of system (1).

**Proposition 7**

Assume that (2) and (3) hold; any solution {*x*_1_(*n*), *x*_2_(*n*), *u*_1_(*n*), *u*_2_(*n*)} of system (1) satisfies

(12) $$\begin{array}{c} \underset{n\to +\infty }{\mathrm{limsup}}\;x_{i}\left(n\right)\leq x_{i}^{*},\;\;\underset{n\to +\infty }{\mathrm{limsup}}\;u_{i}\left(n\right)\leq u_{i}^{*},\;i=1,2, \end{array}$$

where

(13) $$\begin{array}{c} x_{i}^{*}\overset{\mathrm{def}}{=}\frac{\exp\left(r_{i}^{u}-1\right)}{a_{ii}^{l}},\;\;u_{i}^{*}\overset{\mathrm{def}}{=}\frac{f_{i}^{u}\exp\left(r_{i}^{u}-1\right)}{a_{ii}^{l}e_{i}^{l}}. \end{array}$$

**Proof**

Let {*x*_1_(*n*), *x*_2_(*n*), *u*_1_(*n*), *u*_2_(*n*)} be any positive solution of system (1) with initial condition (3). By assumption (2), it follows from the first and second equations of system (1) that

(14) $$\begin{array}{c} x_{i}\left(n+1\right)\leq x_{i}\left(n\right)\exp\left[r_{i}\left(n\right)-a_{ii}\left(n\right)x_{i}\left(n\right)\right],\;i=1,2. \end{array}$$

Applying Lemma 5, one obtains

(15) $$\begin{array}{c} \underset{n\to +\infty }{\mathrm{limsup}}\;x_{i}\left(n\right)\leq \frac{\exp\left(r_{i}^{u}-1\right)}{a_{ii}^{l}}=x_{i}^{*},\;i=1,2. \end{array}$$

Thus, for any *ε* > 0 small enough, it follows from (15) that there exists a large enough integer *n*_1_ such that

(16) $$\begin{array}{c} x_{i}\left(n\right)\leq x_{i}^{*}+\varepsilon \;\text{for}\;\;n\geq n_{1},\;\;i=1,2. \end{array}$$

Then the third and fourth equations of system (1) lead to

(17) ui(n)=∏p=0n−1(1−di(p))[ui(0)+∑p=0n−1ei(p)xi(p)∏q=0n−11−di(q)]  ≤(1−dil)n(ui(0)+vi) +eiu(xi∗+ε)∑p=n0n−1 ∏q=p+1n−1(1−ei(q))≤(1−dil)n(u1(0)+vi)+eiu(xi∗+ε)∑p=n0n−1(1−eil)n−p−1,

where *v*_*i*_ = ∑_*p*=0_^*n*−1^(*e*_*i*_(*p*)*x*_*i*_(*p*)/∏_*q*=0_^*n*−1^1 − *d*_*i*_(*q*)), *i* = 1,2. Since 0 < *d*_1_^*l*^ < 1, we can find a *g* ∈ *ℤ* ^+^ such that 1 − *d*_1_^*l*^ = *e* ^−*g*^; by Stolz's theorem, we have

(18) ∑p=n0n−1(1−eil)n−p−1=∑p=n0n−1eg(p+1)egn⟶11−e−g=1d1l, (n⟶∞).

Thus,

(19) $$\begin{array}{c} \underset{n\to +\infty }{\mathrm{limsup}}\;u_{i}\left(n\right)\leq \frac{\left(x_{i}^{*}+\varepsilon \right)f_{i}^{u}}{e_{i}^{l}}. \end{array}$$

Letting *ε* → 0 and substituting (16) in the above inequality lead to

(20) $$\begin{array}{c} \underset{n\to +\infty }{\mathrm{limsup}}\;u_{i}\left(n\right)\leq \frac{f_{i}^{u}\exp\left(r_{i}^{u}-1\right)}{a_{ii}^{l}e_{i}^{l}}=u_{i}^{*},\;i=1,2. \end{array}$$

**Proposition 8**

Assume that (2) and (3); furthermore

(21) $$\begin{array}{c} r_{i}^{l}\geq a_{ij}^{u}x_{j}^{*}+c_{i}^{u}u_{i}^{*},\\ a_{ii}^{u}+c_{i}^{u}u_{i}^{*}+\left(b_{i}^{u}+a_{ij}^{u}\right)x_{j}^{*}\geq r_{i}^{l}, \end{array}$$

for *i*, *j* = 1,2, *i* ≠ *j*, are satisfied, where *u*_i_* and *x*_i_* are the same as those in Proposition 7. Then

(22) $$\begin{array}{c} \underset{n\to +\infty }{\mathrm{liminf}}\;x_{i}\left(n\right)\geq x_{i^{*}},\;\;\underset{n\to +\infty }{\mathrm{liminf}}\;u_{i}\left(n\right)\geq u_{i^{*}}, \end{array}$$

where

(23) ui∗=defxi∗fileiu,xi∗=defril−aijuxj∗−ciuxi∗aiiu+biuxj∗  ×exp⁡{(ril−aijuxj∗−ciuui∗)−(aiiu+biuxj∗)xi∗}.

**Proof**

For *ε* > 0 and *n* > *n*_2_, according to Proposition 7, there exists a *n*_2_ ∈ *ℤ* ^+^ such that

(24) $$\begin{array}{c} x_{i}\left(n\right)\leq x_{i}^{*}+\varepsilon ,\;\;u_{i}\left(n\right)\leq u_{i}^{*}+\varepsilon . \end{array}$$

By Lemma 6, associating (21) with (1), one has

(25) liminf⁡n→+∞xi(n)≥ril−aiju(xj∗+ε)−ciu(xi∗+ε)aiiu+biu(xj∗+ε)×exp⁡⁡{ril−aiju(xj∗+ε)−ciu(ui∗+ε)−[aiiu+biu(xj∗+ε)]xi∗}.

Letting *ε* → 0 in the above inequality leads to

(26) liminf⁡n→+∞xi(n)≥ril−aijuxj∗−ciuxi∗aiiu+biuxj∗ ×exp⁡{(ril−aijuxj∗−ciuui∗) −(aiiu+biuxj∗)xi∗}=xi∗.

Also from (26), for each *ε* > 0, there exists a large enough integer *n*_0_ such that

(27) $$\begin{array}{c} x_{i}\left(n\right)\geq x_{i^{*}}-\varepsilon \;\;\text{for}\;\;n\geq n_{0},\;\;i=1,2. \end{array}$$

By the third and fourth equations of system (1), we can get that

(28) ui(n)=∏p=0n−1(1−di(p))[ui(0)+∑i=0n−1ei(p)xi(p)∏q=0p1−di(q)]≥(1−diu)n(ui(0)+v1)+eiu(xi∗−ε) ×∑p=n0n−1 ∏q=p+1n−1(1−ei(j))≥(1−diu)n(ui(0)+v1) +eiu(xi∗−ε)∑p=n0n−1(1−eiu)n−p−1,

where *v*_*i*_ = ∑_*p*=0_^*n*−1^(*e*_*i*_(*p*)*x*_*i*_(*p*)/∏_*q*=0_^*n*−1^1 − *d*_*i*_(*q*)), *i* = 1,2. As 0 < *d*_*i*_^*u*^ < 1, similar to the analysis in the proof of Proposition 7, we have

(29) $$\begin{array}{c} \underset{n\to +\infty }{\mathrm{liminf}}u_{i}\left(n\right)\geq \frac{x_{i}^{*}f_{i}^{l}}{e_{i}^{u}}=u_{i^{*}},\;i=1,2. \end{array}$$

Now, we are in a position to state Theorem 9 whose proof is a direct consequence of Propositions 7 and 8.

**Theorem 9**

If the inequalities in (2), (3), and (21) hold, then system (1) is persistent.

## 4. Almost Periodic Solution

Based on Theorem 9, in Section 3, we discuss the almost periodic property for system (1).

Consider the following almost periodic difference system:

(30) $$\begin{array}{c} x\left(n+1\right)=f\left(n,x\left(n\right)\right),\;n\in {\mathbb{Z}}^{+}, \end{array}$$

where *f* : *ℤ* × *S*_*B*_ → ℝ^*k*^, *S*_*B*_ = {*x* ∈ ℝ^*k*^ : ||*x*|| < *B*}, and *f*(*n*, *x*) is almost periodic in *n* uniformly for *x* ∈ *S*_*B*_ and is continuous in *x*. The product system of system (30) is as follows:

(31) $$\begin{array}{c} x\left(n+1\right)=f\left(n,x\left(n\right)\right),\;\;y\left(n+1\right)=f\left(n,y\left(n\right)\right). \end{array}$$

**Lemma 10 (see [2])**

Suppose that there exists a Lyapunov function *V*(*n*, *x*, *y*) defined for *n* ∈ *ℕ*, ||*x*|| < *B*, ||*y*|| < *B* satisfying that

- *a*(||*x* − *y*||) ≤ *V*(*n*, *x*, *y*) ≤ *b*(||*x* − *y*||), where *a*, *b* ∈ *K*, with *K* = {*a* ∈ *C*(ℝ^+^, ℝ^+^) : *a*(0) = 0}, and *a* is increasing;
- |*V*(*n*, *x*_1_, *y*_1_) − *V*(*n*, *x*_2_, *y*_2_)| ≤ *L*(||*x*_1_ − *x*_2_|| + ||*y*_1_ − *y*_2_||), where *L* > 0 is a constant;
- Δ*V*_(10)_(*n*, *x*, *y*)≤−*aV*(*n*, *x*, *y*), where 0 < *a* < 1 is a constant and Δ*V*_(10)_(*n*, *x*, *y*) = *V*(*n* + 1, *f*(*n*, *x*), *f*(*n*, *y*)) − *V*(*n*, *x*, *y*).

Moreover, if there exists a solution *φ*(*n*) of system (30) such that ||*φ*(*n*)||≤B* < *B* for *n* ∈ *ℤ* ^+^, then there exists a unique uniformly asymptotically stable almost periodic solution *p*(*n*) of system (30) which is bounded by B*. In particular, if *f*(*n*, *x*) is periodic of period *ω*, then there exists a unique uniformly asymptotically stable periodic solution of (30) of period *ω*.

According to Lemma 10, we first prove that there exists a bounded solution of system (1) and then construct an adaptive Lyapunov functional for system (1). We denote by *Ω* the set of all solutions *X*(*n*) = {*x*_1_(*n*), *x*_2_(*n*), *u*_1_(*n*), *u*_2_(*n*)} of system (1) satisfying *x*_i*_ ≤ *x*_*i*_(*n*) ≤ *x*_i_*, *u*_i*_ ≤ *u*_*i*_(*n*) ≤ *u*_i_*.

**Lemma 11**

Assume that (2), (3), and the conditions of Theorem 9 hold; then *Ω* ≠ *Ø*.

**Proof**

It is now possible to show by an inductive argument that the system (1) leads to

(32) xi(n)=xi(0)exp⁡∑l=0n−1[ri(l)−aii(l)xi(l)−aij(l)xj(l)−bi(l)xi(l)xj(l)−ci(l)ui(l)],ui(n)=ui(0)−∑l=0n−1{di(l)ui(l)−ei(l)xi(l)},

for *i*, *j* = 1,2, *i* ≠ *j*. From Theorem 9, for any solution *X*(*n*) = {*x*_1_(*n*), *x*_2_(*n*), *u*_1_(*n*), *u*_2_(*n*)} of system (1) with initial condition (2) satisfies Definition 1. Hence, for any *ε* > 0, there exists a *n*_0_; if *n*_0_ is sufficiently large, we have

(33) xi∗−ε≤xi(n)≤xi∗+ε,  ui∗−ε≤ui(n)≤ui∗+ε,i=1,2.

Let {*τ*_*α*_} be any integer valued sequence such that *τ*_*α*_ → *∞* as *α* → *∞*; we claim that there exists a subsequence of {*τ*_*α*_}, and we still denote it by {*τ*_*α*_}, such that *x*_*i*_(*n* + *τ*_*α*_) → *x*_i_*(*n*) uniformly in *n* on any finite subset *B* of *ℤ* as *α* → *∞*, where

(34) $$\begin{array}{c} B=\left\{a_{1},a_{2},\ldots ,a_{m}\right\},\;\;a_{h}\in \mathbb{Z},\;\;h=\left(1,2,\ldots ,m\right), \end{array}$$

and *m* is a finite number.

In fact, for any finite subset *B* ⊂ *ℤ*, when *α* is large enough, *τ*_*α*_ + *a*_*α*_ > *n*_0_, *h* = 1,2,…, *m*. So

(35) $$\begin{array}{c} x_{i^{*}}-\varepsilon \leq x_{i}\left(n+\tau_{\alpha }\right)\leq x_{i}^{*}+\varepsilon ,\\ u_{i^{*}}-\varepsilon \leq u_{i}\left(n+\tau_{\alpha }\right)\leq u_{i}^{*}+\varepsilon . \end{array}$$

That is, {*x*_*i*_(*k* + *τ*_*α*_), *u*_*i*_(*k* + *τ*_*α*_)} are uniformly bounded for large enough *α*.

Now, for *a*_1_ ∈ *B*, we can choose a subsequence *τ*_*α*_^(1)^ of {*τ*_*α*_} such that {*x*_*i*_(*a*_1_ + *τ*_*α*_^(1)^), *u*_*i*_(*a*_1_ + *τ*_*α*_^(1)^)} uniformly converges on *ℤ* ^+^ for *α* large enough.

Similarly, for *a*_2_ ∈ *B*, we can choose a subsequence *τ*_*α*_^(2)^ of {*τ*_*α*_} such that {*x*_*i*_(*a*_2_ + *τ*_*α*_^(2)^), *u*_*i*_(*a*_2_ + *τ*_*α*_^(2)^)} uniformly converges on *ℤ* ^+^ for *α* large enough.

Repeating this procedure, for *a*_*m*_ ∈ *B*, we can choose a subsequence {*τ*_*α*_^(*m*−1)^} of {*τ*_*α*_^(*m*)^} such that {*x*_*i*_(*a*_*m*_ + *τ*_*α*_^(*m*)^), *u*_*i*_(*a*_*m*_ + *τ*_*α*_^(*m*)^)} uniformly converges on *ℤ* ^+^ for *α* large enough.

Now pick the sequence {*τ*_*α*_^(*m*)^} which is a subsequence of {*τ*_*α*_}; we still denote it by {*τ*_*α*_}; then for all *k* ∈ *B*, we have

(36) $$\begin{array}{c} \left\{x_{i}\left(n+\tau_{\alpha }\right)⟶x_{i}^{*},u_{i}\left(n+\tau_{\alpha }\right)⟶u_{i}^{*}\right\} \end{array}$$

uniformly in *k* ∈ *B* as *α* → *∞*.

By the arbitrary of *B*, the conclusion is valid.

Since {*r*_*i*_(*n*)}, {*a*_*ij*_(*n*)}, {*b*_*i*_(*n*)}, {*c*_*i*_(*n*)}, {*d*_*i*_(*n*)}, and {*e*_*i*_(*n*)} are almost periodic sequence, for the above sequence *τ*_*α*_, *τ*_*α*_ → *∞* as *α* → *∞*, there exists a subsequence which we still denote it by {*τ*_*α*_} (if necessary, we take subsequence), such that

(37) $$\begin{array}{c} r_{i}\left(n+\tau_{\alpha }\right)⟶r_{i}\left(n\right),\;\;a_{ij}\left(n+\tau_{\alpha }\right)⟶r_{i}\left(n\right),\\ b_{i}\left(n+\tau_{\alpha }\right)⟶b_{i}\left(n\right),\;\;c_{i}\left(n+\tau_{\alpha }\right)⟶c_{i}\left(n\right),\\ \;\;d_{i}\left(n+\tau_{\alpha }\right)⟶d_{i}\left(n\right),\;\;e_{i}\left(n+\tau_{\alpha }\right)⟶e_{i}\left(n\right), \end{array}$$

as *α* → *∞* uniformly on *ℤ* ^+^.

For any *δ* ∈ *ℤ*, we can assume that *τ*_*α*_ + *δ* ≥ *n*_0_ for *δ* large enough. Letting  *n* ≥ 0 and *n* ∈ *ℤ*, by an inductive argument of (1) from *τ*_*α*_ + *δ* to *n* + *τ*_*α*_ + *δ*, leads to

(38) xi(n+τα+δ)=xi(τα+δ) ×exp⁡∑l=τα+δn+τα+δ−1[ri(l)−aii(l)xi(l)−aij(l)xj(l)−bi(l)xi(l)xj(l)−ci(l)ui(l)],ui(n+τα+δ)=ui(τα+δ)−∑l=ταn+τα+δ−1{di(l)ui(l)−ei(l)xi(l)}.

Then, for *i*, *j* = 1,2, *i* ≠ *j*, we have

(39) xi(n+τα+δ)=xi(τα+δ)exp⁡∑l=δn+δ−1[ri(l+τα)−aii(l+τα)xi(l+τα)−aij(l+τα)xj(l+τα)−bi(l+τα)xi(l+τα)xj(l+τα)−ci(l+τα)ui(l+τα)],ui(n+τα+δ)=ui(τα+δ)−∑l=δn+δ−1{di(l+τα)ui(l+τα)−ei(l+τα)xi(l+τα)}.

Let *α* → *∞*; for any *k* ≥ 0,

(40) xi∗(n+δ)=xi(δ)exp⁡∑l=δn+δ−1[ri(l)−aii(l)xi(l)−aij(l)xj(l)−bi(l)xi(l)xj(l)−ci(l)ui(l)],ui∗(n+δ)=ui(δ)−∑l=δn+δ−1{di(l)ui(l)−ei(l)xi(l)}.

By the arbitrariness of *δ*, X*(*n*) = {*x*_1_*(*n*), *x*_2_*(*n*), *u*_1_*(*n*), *u*_2_*(*n*)} is a solution of system (1) on *ℤ* ^+^. It is clear that 0 ≤ *x*_i*_ ≤ *x*_*i*_(*n*) ≤ *x*_i_*; 0 ≤ *u*_i*_ ≤ *u*_*i*_(*n*) ≤ *u*_i_*, *n* ∈ *ℤ* ^+^.

So *Ω* ≠ Φ. Lemma 10 is valid.

Before stating Theorem 12, for the sake of convenience, we set

(41) rij=2(aiil+bilxi∗)−[(aiiu+biuxj∗)2+ei2u]xi∗2 −[ajiu+bjuxj∗]2xi∗2+(aiju+biuxi∗)+(ajiu+bjuxj∗) −(aiiu+biuxj∗)(aiju+biuxi∗)xi∗xj∗ −(ajju+bjuxi∗)(ajiu+bjuxj∗)xi∗xj∗, +cil(aiil+bilxj∗)xi∗−ciu−(1−diL)eiuxi∗ −cju(ajiu+biuxi∗)xi∗,rij∗=−ciu−(1−dil)eiuxi∗+cil(aiil+bilxj∗)xi∗ −ciu(aiju+bjuxi∗)xj∗−di2u+2dil−ciu.

**Theorem 12**

Suppose that the conditions of Lemma 10 are satisfied; moreover, 0 < *β* < 1, where

(42) $$\begin{array}{c} \beta =\min\left\{r_{ij},r_{ij}^{*}\right\},\;\text{for}\;\;i,j=1,2,\;\;i\neq j. \end{array}$$

Then there exists a unique uniformly asymptotically stable almost periodic solution *X*(*n*) = {*x*_1_(*n*), *x*_2_(*n*), *u*_1_(*n*), *u*_2_(*n*)} of system (1) which is bounded by *Ω* for all *n* ∈ *ℤ* ^+^.

**Proof**

Let *x*_*i*_(*n*) = exp⁡*p*_*i*_(*n*); from system (1), we have

(43) pi(n+1)=pi(n)+ri(n)−aii(n)exp⁡pi(n) −aij(n)exp⁡pj(n)−bi(n)exp⁡pi(n)exp⁡pj(n) −ci(n)ui(n),Δui(n)=−di(n)ui(n)+ei(n)exp⁡pi(n),

where *i*, *j* = 1,2, *i* ≠ *j*. From Lemma 10, it shows that system (43) has a bounded solution *Y*(*n*) = {*p*_1_(*n*), *p*_2_(*n*), *u*_1_(*n*), *u*_2_(*n*)} satisfying

(44) ln⁡xi∗≤pi(n)<ln⁡xi∗,  ui∗≤ui(n)≤ui∗,i=1,2,  n∈ℤ+.

Thus,

(45)        |pi(n)|≤Ai,  |ui(n)|≤Bi,Ai=max⁡⁡{|ln⁡xi∗|,|ln⁡xi∗|},  Bi=max⁡⁡{ui∗,ui∗},                        i=1,2.

For (*X*, *U*) ∈ ℝ^2+2^, we define a norm

(46) $$\begin{array}{c} ||\left(X,U\right)||=\sum_{i=1}^{2}\left|x_{i}\right|+\sum_{i=1}^{2}\left|u_{i}\right|. \end{array}$$

For the product form of system (43)

(47) pi(n+1)=pi(n)+ri(n)−aii(n)exp⁡pi(n) −aij(n)exp⁡pj(n)−bi(n)exp⁡pi(n)exp⁡pj(n) −ci(n)ui(n),Δui(n)=−di(n)ui(n)+ei(n)exp⁡pi(n),qi(n+1)=qi(n)+ri(n)−aii(n)exp⁡qi(n) −aij(n)exp⁡qj(n)−bi(n)exp⁡qi(n)exp⁡qj(n) −ci(n)vi(n),Δvi(n)=−di(n)vi(n)+ei(n)exp⁡qi(n).

Let *Z* = {*p*_1_(*n*), *p*_2_(*n*), *u*_1_(*n*), *u*_2_(*n*)} and *W* = {*q*_1_(*n*), *q*_2_(*n*), *v*_1_(*n*), *v*_2_(*n*)} be any two solutions of system (30) defined on *ℤ* ^+^ × S* × S*; then ||*Z*|| ≤ *B* and ||*W*|| ≤ *B*, where *B* = ∑_*i*=1_^2^{*A*_*i*_ + *B*_*i*_}, and

(48) S∗={Y(n) ∣ ln⁡xi∗≤pi(n)≤ln⁡xi∗,ui∗≤ui(n)≤ui∗, i=1,2}.

For convenience, we denote

(49) $$\begin{array}{c} M_{i}\left(n\right)=p_{i}\left(n\right)-q_{i}\left(n\right),\;\;N_{i}\left(n\right)=u_{i}\left(n\right)-v_{i}\left(n\right),\\ {\tilde{M}}_{i}\left(n\right)={\tilde{p}}_{i}\left(n\right)-{\tilde{q}}_{i}\left(n\right),\;\;{\tilde{N}}_{i}\left(n\right)={\tilde{u}}_{i}\left(n\right)-{\tilde{v}}_{i}\left(n\right). \end{array}$$

Let

(50) $$\begin{array}{c} V\left(n,Z,W\right)=\sum_{i=1}^{2}M_{i}^{2}\left(n\right)+N_{i}^{2}\left(n\right) \end{array}$$

be a function on *ℤ* ^+^ × S* × S*.

Then

(51) $$\begin{array}{c} ||Z-W||=\sum_{i=1}^{2}\left\{\left|M_{i}\left(n\right)\right|+\left|N_{i}\left(n\right)\right|\right\},\\ {||Z-W||}_{*}={\left\{\sum_{i=1}^{2}\left\{M_{i}^{2}(n)+N_{i}^{2}(n)\right\}\right\}}^{1/2} \end{array}$$

are equivalent. It is easy to see that

(52) $$\begin{array}{c} {\left(C_{1}||Z-W||\right)}^{2}\leq V\left(n,Z,W\right)\leq {\left(C_{2}||Z-W||\right)}^{2}. \end{array}$$

Let *a* ∈ *C*(ℝ^+^, ℝ^+^), *a*(*x*) = *C*_1_^2^ *x* ^2^, *b* ∈ *C*(ℝ^+^, ℝ^+^), and *b*(*x*) = *C*_2_^2^ *x* ^2^; thus condition (i) in Lemma 10 is satisfied.

In addition,

(53) |V(n,Z,W)−V(n,Z~,W~)|=|∑i=12(Mi2+Ni2)−∑i=12(M~i2+N~i2)|≤∑i=12{|Mi(n)+M~i(n)|·|Mi(n)−M~i(n)|} +∑i=12{|Ni(n)+N~i(n)|·|Ni(n)−N~i(n)|}≤L{∑i=12(|Mi(n)|+|M~i(n)|)+∑i=12(|Ni(n)|+|N~i(n)|)}=L{||Z−Z~||+||W−W~||},

where *L* = 4max⁡{*A*_*i*_, *B*_*i*_}, *i* = 1,2. And Lemma 10 (ii) is held.

Finally, calculating the Δ*V* along the solutions of (47) leads to

(54) ΔV(12)(n)=∑i=12{Mi+12(n)+Ni+12(n)}−∑i=12{Mi2(n)+Ni2(n)}=∑i=12[(aii(n)+bi(n)epj(n))2+ei2(n)](epi(n)−eqi(n))2 +[aij(n)+bi(n)eqi(n)]2(epj(n)−eqj(n))2 +2[aii(n)+bi(n)epj(n)] ·[aij(n)+bi(n)eqi(n)](epi(n)−eqi(n)) ×(epj(n)−eqj(n))−2Mi(n) ·[aii(n)+bi(n)epj(n)](epi(n)−eqi(n)) −2Mi(n)[aij(n)+bi(n)eqj(n)] ·(epj(n)−eqj(n))+2ciMi(n)Ni(n) +2[(1−di(n))ei(n)+ci(n)·(aii(n)+bi(n)epj(n))]Ni(n)(epi(n)−eqi(n)) +2ci[aij(n)+bi(n)eqi(n)] ·Ni(n)(epj(n)−eqj(n))+[di2(n)−2di(n)+ci(n)]2Ni2(n).

By the mean value theorem we get *e* ^*p*_*i*_(*n*)^ − *e* ^*q*_*i*_(*n*)^ = *ξ*(*n*)*M*_*i*_(*n*), where *ξ*(*n*) lies between *e* ^*p*_*i*_(*n*)^ and *e* ^*q*_*i*_(*n*)^.

From (49), (54), one has

(55) ΔV(12)(n)=∑i=12{[(aii(n)+bi(n)epj(n))2+ei2(n)]×ξi2[(pi(n)−qi(n)]2+[aij(n)+bi(n)eqi(n)]2ξj2[pj(n)−qj(n)]2+2[aii(n)+bi(n)epj(n)][aij(n)+bi(n)eqi(n)]·ξiξj[pi(n)−qi(n)][pj(n)−qj(n)]−2[pi(n)−qi(n)][aii(n)+bi(n)epj(n)]×ξi[pi(n)−qi(n)]+2[pi(n)−qi(n)][aij(n)+bi(n)eqj(n)]×ξj[pj(n)−qj(n)]+2ci[pi(n)−qi(n)][ui(n)−vi(n)]+2[(1−di(n))ei(n)+ci(n)(aii(n)+bi(n)epj(n))]·(ui(n)−vi(n))ξi[pi(n)−qi(n)]+2ci[aij(n)+bi(n)eqi(n)](ui(n)−vi(n))fffffff×ξj[pj(n)−qj(n)]+[di2(n)−2di(n)+ci(n)]2(ui(n)−vi(n))2}=∑i=12{{[(aii(n)+bi(n)epj(n))2+ei2(n)]ξi2−2[aii(n)+bi(n)epj(n)ξ]}·Mi2(n)+[aij(n)+bi(n)eqi(n)]2ξj2Mj2(n)+(2[aii(n)+bi(n)epj(n)][aij(n)+bi(n)eqi(n)]ξiξj−2[aij(n)+bi(n)·eqj(n)]ξj)Mi(n)Mj(n)+[di2(n)−2di(n)+ci(n)]2Ni2(n)+2[ci+[(1−di(n))ei(n)+ci(n)(aii(n)+bi(n)epj(n))]ξi]×Mi(n)Ni(n)+2ci[aij(n)+bi(n)eqi(n)]×Ni(n)ξj[pj(n)−qj(n)]}.

We get for *j* = 1,2 that

(56) $$\begin{array}{c} \Delta V_{\left(12\right)}\left(n\right)\leq \sum_{i=1}^{2}\left\{{V_{1}}_{ij}+{V_{2}}_{ij}+{V_{3}}_{ij}+{V_{4}}_{ij}+{V_{5}}_{ij}+{V_{6}}_{ij}\right\}, \end{array}$$

where

(57) V1ij={[(aii(n)+bi(n)epj(n))2+ei2(n)]ξi2−2[aii(n)+bi(n)epj(n)ξi]}Mi2(n)≤{(aiiu+biuxj∗)2+ei2u)xi∗2−2(aiil+bilxi∗)xi∗}Mi2(n);V2ij={[aij(n)+bi(n)eqi(n)]2ξj2}Mj2(n),≤{[aiju+biuxi∗]2xj∗2}Mj2(n);V3ij={2[aii(n)+bi(n)epj(n)][aij(n)+bi(n)eqi(n)]ξiξj−2[aij(n)+bi(n)eqj(n)]ξj}Mi(n)Mj(n)≤{(aiiu+biuxj∗)(aiju+biuxi∗)xi∗xj∗−(aiju+biuxi∗)} ×[Mi2(n)+Mj2(n)];V4ij={2ci+2[(1−di(n))ei(n)+ci(n)(aii(n)+bi(n)epj(n))]ξi} ×Mi(n)Ni(n)≤{ciu+[(1−dil)eiu+cil(aiil+bilxj∗)]xi∗} ×[Mi2(n)+Ni2(n)];V5ij={2ci[aij(n)+bj(n)eqi(n)]ξj}Mj(n)Ni(n)≤{ciu(aiju+bjuxi∗)xj∗}[Mj2(n)+Ni2(n)];V6ij={[di2(n)−2di(n)+ci(n)]2}Ni2(n)≤{di2u−2dil+ciu}Ni2(n).

Hence,

(58) ΔV(12)(n)≤∑i=12{[[(aiiu+biuxj∗)2+ei2u]xi∗2−2(aiil+bilxi∗)xi∗]·[(pi(n)−qi(n)]2+[[aiju+biuxi∗]2xj∗2]Mj2(n)+[(aiiu+biuxj∗)(aiju+biuxi∗)xi∗xj∗−(aiju+biuxi∗)]×Mi2(n)Mj2(n)+[ciu+[(1−dil)eiu−cil(aiil+bilxj∗)]xi∗]×Mi2(n)Ni2(n)+[ciu(aiju+biuxi∗)xj∗][Mj2(n)+Ni2(n)]+[di2u−2dil+ciu]Ni2(n)}=−∑i=12{[2(aiil+bilxi∗)−[(aiiu+biuxj∗)2+ei2u]xi∗2−[ajiu+bjuxj∗]2xi∗2+(aiju+biuxi∗)+(ajiu+bjuxj∗)−(aiiu+biuxj∗)(aiju+biuxi∗)xi∗xj∗−(ajju+bjuxi∗)(ajiu+bjuxj∗)xi∗xj∗+cil(aiil+bilxj∗)xi∗−ciu−(1−dil)eiuxi∗−cju(ajiu+biuxi∗)xi∗]·Mi2(n)+[−ciu−(1−dil)eiuxi∗+cil(aiil+bilxj∗)xi∗−ciu(aiju+biuxi∗)xj∗−di2u+2dil−ciu]Ni2(n)}≤−∑i=12[rijMi2(n)+rij∗Ni2(n)]≤−β∑i=12[Mi2(n)+Ni2(n)]≤−βV(n),

where *β* = min⁡{*r*_*ij*_, *r*_ij_*}, *i*, *j* = 1,2, *i* ≠ *j*. That is, there exists a positive constant 0 < *β* < 1 such that

(59) $$\begin{array}{c} \Delta V_{\left(12\right)}\left(n\right)\leq -\beta V\left(n\right). \end{array}$$

From 0 < *β* < 1, condition (iii) of Lemma 10 is satisfied. So, from Lemma 10, there exists a uniqueness uniformly asymptotically stable almost periodic solution *X*(*n*) = {*x*_1_(*n*), *x*_2_(*n*), *u*_1_(*n*), *u*_2_(*n*)} of system (43) which is bounded by S* for all *n* ∈ *ℤ* ^+^, which means that there exists a uniqueness uniformly asymptotically stable almost periodic solution *X*(*n*) = {*x*_1_(*n*), *x*_2_(*n*), *u*_1_(*n*), *u*_2_(*n*)} of system (1) which is bounded by *Ω* for all *n* ∈ *ℤ* ^+^. This completes the proof.
